# Rhythm Analysis during Cardiopulmonary Resuscitation: Past, Present, and Future

**DOI:** 10.1155/2014/386010

**Published:** 2014-01-09

**Authors:** Sofia Ruiz de Gauna, Unai Irusta, Jesus Ruiz, Unai Ayala, Elisabete Aramendi, Trygve Eftestøl

**Affiliations:** ^1^Communications Engineering Department, University of the Basque Country (UPV/EHU), Alameda Urquijo S/N, 48013 Bilbao, Spain; ^2^Department of Electrical Engineering and Computer Science, Faculty of Science and Technology, University of Stavanger, 4036 Stavanger, Norway

## Abstract

Survival from out-of-hospital cardiac arrest depends largely on two factors: early cardiopulmonary resuscitation (CPR) and early defibrillation. CPR must be interrupted for a reliable automated rhythm analysis because chest compressions induce artifacts in the ECG. Unfortunately, interrupting CPR adversely affects survival. In the last twenty years, research has been focused on designing methods for analysis of ECG during chest compressions. Most approaches are based either on adaptive filters to remove the CPR artifact or on robust algorithms which directly diagnose the corrupted ECG. In general, all the methods report low specificity values when tested on short ECG segments, but how to evaluate the real impact on CPR delivery of continuous rhythm analysis during CPR is still unknown. Recently, researchers have proposed a new methodology to measure this impact. Moreover, new strategies for fast rhythm analysis during ventilation pauses or high-specificity algorithms have been reported. Our objective is to present a thorough review of the field as the starting point for these late developments and to underline the open questions and future lines of research to be explored in the following years.

## 1. Introduction

In the early 1990s, the American Heart Association (AHA) established the chain of survival [1] to describe the sequence of actions for a successful resuscitation in the event of an out-of-hospital cardiac arrest (OHCA). The chain of survival involves four links: early recognition, early bystander cardiopulmonary resuscitation (CPR), early defibrillation, and early advanced care. The most influential factor explaining survival is the interaction between CPR and defibrillation administered in the first minutes from collapse [2]. Survival from witnessed ventricular fibrillation (VF) decreases by 10–12% for every minute defibrillation is delayed [3, 4], but when CPR is provided the decline in survival is only 3-4% per minute [4–6]. CPR and defibrillation can be successfully taught to laypeople, and the use of automated external defibrillators (AED) by the public may shorten the time to defibrillation [7].

Over the years, evidence has accumulated suggesting that minimizing the interruptions in chest compressions during CPR is determinant for survival from OHCA [8–11]. Consequently, current resuscitation guidelines emphasize the importance of high-quality CPR with minimal interruptions in chest compressions [12, 13]. However, CPR must be interrupted for a reliable AED rhythm analysis. The mechanical activity from the chest compressions introduces artifacts in the ECG that substantially lower the capacity of an AED's shock advice algorithm (SAA) to detect shockable (sensitivity) and nonshockable (specificity) rhythms [14, 15]. Interruptions for rhythm analysis alone take between 5.2 s and 28.4 s in commercial AEDs [16]. These interruptions, known as hands-off intervals, adversely affect the probability of restoration of spontaneous circulation (ROSC) after the delivery of the shock [17] and compromise circulation [18]. In fact, a recent multicenter study found an 18% decrease in survival to hospital discharge for every 5 s increase in preshock pause length [19]. Therefore, reliable rhythm analysis methods during chest compressions would be of great value.

Over the last 15 years, many efforts have been made to reliably analyze the rhythm during CPR. Strategies have focused either on adaptive filters to suppress the CPR artifact [20] or, more recently, on approaches based on the direct analysis of the corrupted ECG. Most studies report sensitivities above 90%, the minimum value recommended by the AHA for AED performance [21]. However, the specificity rarely exceeds 85%, well below the 95% AHA goal. As Li and Tang phrased it back in 2009, performance is *good but not enough* [22]. In addition, the impact these methods would have on CPR delivery is unknown. The current evaluation standard is based on the sensitivity and specificity of a single analysis using short duration (10–20 s) segments. This does not reflect the real application scenario in which the objective would be to continuously analyze the rhythm during CPR. In this context, the fundamental question is whether rhythm analysis improves CPR delivery compared to the standard treatment, that is, cycles of 2 minutes of uninterrupted CPR followed by a hands-off interval for rhythm assessment. This change of focus was stressed by the International Consensus on CPR and Emergency Cardiovascular Care Science with Treatment Recommendations (CoSTR) in 2010 [23].

Recent developments preclude the start of a new era in the field of rhythm analysis during CPR. A new methodology has just been developed to measure the impact of continuous rhythm analysis on CPR delivery [24]. In addition, new ideas have been explored, like the possibility of assessing the rhythm during ventilation pauses [25] using SAAs capable of diagnosing the rhythm in less than 5 s [26]. At this point a review paper that goes beyond the compilation and summary of filtering methods is well justified. Our objective is to present a thorough review of the field as the starting point for these late developments and to underline the open questions and future lines of research to be explored in the coming years.

The paper is structured as follows.  Section 2 describes the characteristics of the CPR artifact and presents the problem of rhythm analysis during CPR.  Section 3 is a review of the approaches to rhythm analysis during CPR up to year 2012, grouped by the evaluation methodology.  Section 4 describes a new methodology to quantify the impact on CPR delivery of rhythm analysis during chest compressions.  Section 5 presents the late developments in rhythm analysis during CPR.

## 2. Context

**Figure 1:**
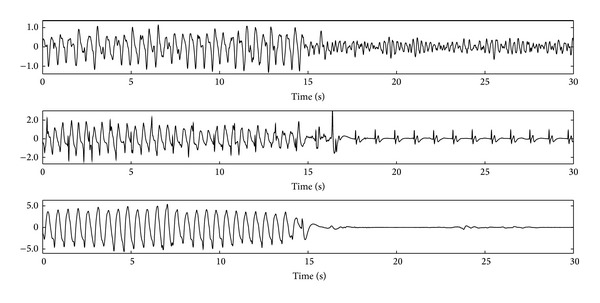
ECG segments in mV recorded in patients in OHCA. The top panel shows a VF, the middle panel shows a PEA, and the bottom panel shows an asystole. In all cases CPR artifacts corrupt the ECG in the initial 15 s interval. In the second 15 s interval chest compressions were stopped and the ECG shows the underlying rhythm.

Chest compressions introduce an artifact in the ECG that substantially modifies its waveform. For example, Figure 1 shows three OHCA segments where CPR corrupts the ECG during the first 15 s of the segment. During the last 15 s chest compressions ceased, revealing the underlying rhythms: VF, pulseless electrical activity (PEA), and asystole. During CPR, the artifact sometimes resembles a regular rhythm with rates around 100 compression per minute (cpm). In these case the AED may give a wrong no shock diagnosis if the underlying rhythm is shockable, that is, VF or fast ventricular tachycardia (VT). Conversely, chest compression artifacts may also introduce fast and disorganized artifacts which might cause an erroneous shock diagnosis if the underlying rhythm is nonshockable. Consequently, the accuracy of commercial AEDs substantially decreases in the presence of CPR artifacts. For example, sensitivity/specificity values of 58.4%/90.8% and 81.5%/67.2% have been reported [14, 15], although these figures are extremely dependent on the design characteristics of each SAA.

The origin of the CPR artifact is not fully understood. Langhelle et al. [32] conjectured that the CPR artifact is an additive noise and identified four possible sources for the artifact: the mechanical stimulation of the heart, the mechanical stimulation of the thoracic muscles, electrode tapping or dragging, and static electricity. Later, Fitzgibbon et al. [33] experimentally concluded that the main source of noise was the skin-electrode interface, specifically, that the noise was related to the electrical properties of the electrode. When chest compressions are delivered manually the characteristics of the artifact are very variable and depend on how the compressions are administered (rate, depth, and pauses) and on the characteristics of both the patient and the recording system.

**Figure 2:**
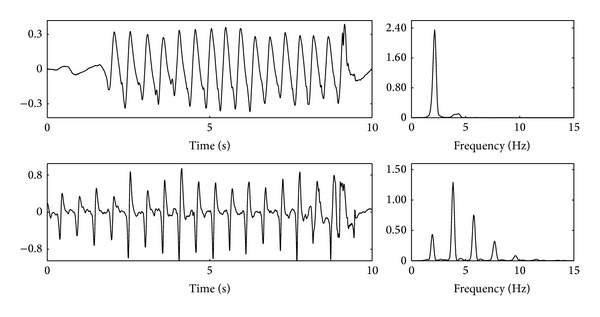
Two examples in the time and frequency domain of CPR artifacts recorded in OHCA patients in asystole. The figures show the ECG in mV and the normalized power spectral density (PSD) in the frequency domain. The first example has pauses in chest compressions, a rate of 133 cpm (2.22 Hz), and small harmonic content. The second example has no pauses, a rate of 116 cpm (1.93 Hz), and large harmonic content.

**Figure 3:**
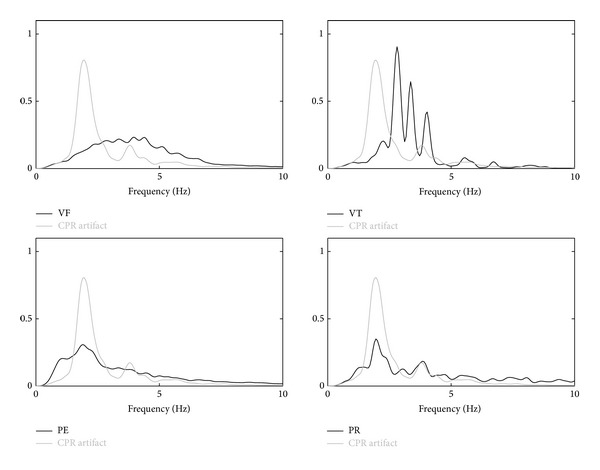
Normalized PSD of CPR artifacts (patients in asystole) and rhythms recorded during OHCA. The spectral overlap is large for both shockable (VF and VT) and nonshockable rhythms (PEA and PR).

The nature of the CPR artifact is best analyzed when CPR is performed on patients in asystole (no underlying heart rhythm) because the ECG only reflects the presence of the artifact, as shown in the last example of Figure 1. The artifact presents an almost periodic waveform, with its fundamental frequency being that of the chest compressions. However, the waveform and spectral characteristics of the artifact are very variable within a resuscitation episode and between episodes. Within an episode these variations may reflect changes on how CPR is administered by a rescuer, rescuer fatigue, or the intervention of several rescuers. For example, Figure 2 shows two short segments of CPR artifacts with very different waveforms and spectral content. In addition to its interpatient and interrescuer variability, on average the artifact presents an important spectral overlap with human ECG recorded during cardiac arrest. This is best seen by analyzing the power spectral density (PSD) of the CPR artifact and the different OHCA rhythms, as shown in Figure 3 for shockable (VF and VT) and nonshockable (PEA and pulse-giving rhythm, PR) rhythms. As shown in the figure the overlap is specially large for nonshockable rhythms, which anticipates the challenge of rhythm analysis during CPR for underlying nonshockable rhythms.

**Figure 4:**
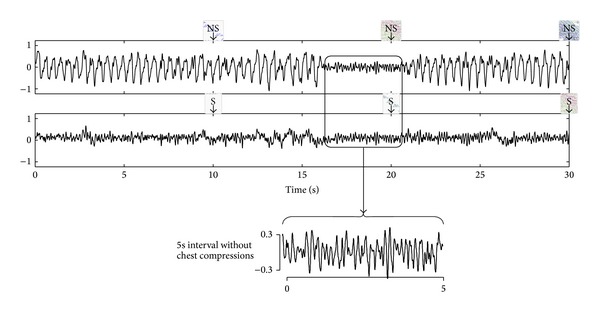
Filtering example for a VF recorded during OHCA. In the top panel the ECG is corrupted by CPR artifacts; a SAA from a commercial AED analyzes the rhythm every 10 s and gives erroneous no-shock (NS) diagnoses. In the bottom panel the CPR artifact is suppressed using an adaptive filter, the underlying VF is revealed, and the SAA gives correct shock (S) diagnoses. The underlying VF is visible in the 5 s interval without chest compressions.

In conclusion, a reliable rhythm analysis during CPR involves advanced signal processing techniques to address the time-frequency variability of the artifact and its spectral overlap with human OHCA rhythms. These techniques are described in the following section. To conclude, Figure 4 illustrates the use of an adaptive filter for rhythm analysis during CPR. In the top panel of the figure the underlying VF is corrupted by CPR artifacts, although it is visible in the 5 s interval without chest compressions. The artifacts provoke erroneous no-shock diagnoses by an AED. Applying an adaptive filter reveals the underlying VF, and the AED correctly diagnoses the rhythm as shockable.

## 3. Overview of Rhythm Analysis during CPR

Research on the suppression of the CPR artifact started in the mid 1990s within the field of VF waveform analysis. VF waveform analysis for shock outcome prediction is beyond the scope of this paper; excellent reviews of this topic are available in the literature [34, 35]. In the first study by Strohmenger et al. [36] and in subsequent ones [37, 38], VF was induced in pigs and chest compressions were administered using a pneumatic piston at a constant chest compression rate of 80 cpm (1.33 Hz). The CPR artifact was successfully removed using digital high-pass filters with cut-off frequencies between 4 and 4.5 Hz [37, 38], because the dominant frequency of VF is around 9–11 Hz in pigs. However, in the human case VF dominant frequencies fall between 3 and 5 Hz [39], the spectral overlap with the CPR artifact is large, and the artifact cannot be removed using a simple high-pass filter [32, 39].

Given the characteristics of the CPR artifact, suppressing it from the human ECG requires adaptive filters, most of which use reference signals correlated with the artifact. Reference signals such as the thoracic impedance, the compression depth, or the compression force have been frequently used. Over the years many adaptive solutions have been proposed and evaluated. The methodology followed in these studies depended largely on the data available to the researchers. Studies can be grouped into two broad categories: those based on the artificial mixture of ECG data and CPR artifacts and those based on cardiac arrest data recorded during CPR.

### 3.1. Studies Based on Artificial Mixtures

The mixture model was introduced early in 2000 by Langhelle et al. [32] and Aase et al. [40]. This model assumes that the CPR artifact, *s*_cpr_, is an additive noise independent of the underlying ECG, *s*_ecg_. Based on this assumption, filtering methods can be tested using independently recorded human ECG and CPR artifacts, added at different signal-to-noise ratios (SNRs) according to

(1) $$\begin{array}{c} s_{\text{cor}}=s_{\text{ecg}}+\alpha_{\text{SNR}}\cdot s_{\text{cpr}},\;\text{with}\;\alpha_{\text{SNR}}=\sqrt{\frac{P_{\text{ecg}}}{P_{\text{cpr}}\cdot 10^{\text{SNR}/10}}}. \end{array}$$

**Figure 5:**
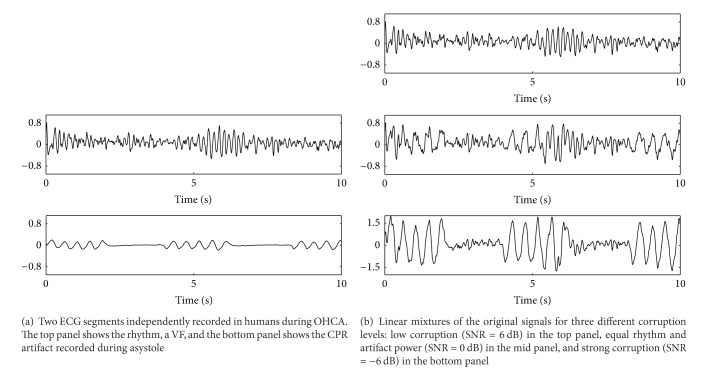
The mixture model: combination of a human VF and a human CPR artifact recorded from a patient in asystole at different SNR.

The SNR in dB is adjusted in the artificial mixture, *s*_cor_, using the *α*_SNR_ coefficient, where *P*_ecg_ and *P*_cpr_ are the power of the underlying ECG and the CPR artifact, respectively.  Figure 5 shows an example of how a human VF and a CPR artifact are combined when the additive model is used.

Typically these mixtures are formed with SNR values in the −10 dB (strong corruption) to 10 dB (low corruption) range. CPR artifacts are recorded during asystole, together with the reference signals used by the adaptive filters to model the artifact. The corrupted signal is fed to the filter which estimates the underlying ECG, and the estimated and the original ECGs are compared to quantify the efficiency of the filter in terms of the improvement of the SNR after filtering [32, 40]. In addition, the clinical accuracy of the method can be assessed using the filtered ECG to evaluate the sensitivity and specificity of an AED's SAA.

Langhelle et al. combined 25 human VF with CPR artifacts recorded from one pig, with CPR delivered by a mechanical device at a constant rate of 90 cpm (1.5 Hz). Their conjugate gradient adaptive filter could only use one reference channel besides the ECG (dual-channel methods), and the best filtering results were obtained for a reference that combined the thoracic impedance and the chest displacement measured at the mechanical device. Furthermore, when compared to a high-pass filter with 4.9 Hz cut-off frequency, their adaptive solution presented higher SNR improvement, with differences of up to 10 dB for low corruption levels. Aase et al. combined 200 human VF and 71 VT with CPR artifacts obtained from two pigs, with CPR delivered by a mechanical device at rates of 60, 90, and 120 cpm (1, 1.5, and 2 Hz). Although their Wiener filter could use an arbitrary number of reference signals (multichannel methods), they used only two: the thoracic impedance acquired via the defibrillation pads and the chest displacement. Not only they did optimize and test their method in terms of how filtering improved the SNR, but also they were the first to report the sensitivity of a SAA after filtering. They showed that the SNR after filtering was lower for higher compression rates (120 cpm) due to the increased spectral overlap and that filtering improved the sensitivity for low SNR. These results were extended by Husøy et al. [41] using the same human data combined with CPR artifacts recorded from pigs. This time CPR was delivered manually at 120 cpm rate, which reflects better the variability of the artifact found in real cardiac arrest episodes. The compression depth was calculated in this study from an external accelerometer based device [42]. Their Multichannel Recursive Adaptive Matching Pursuit (MC-RAMP) filter substantially lowered the computational demands of the Wiener filter and yielded comparable SNR results after filtering.

In a set of complementary studies, a group of Austrian researchers analyzed various dual-channel methods. They used an invasive arterial blood pressure signal as the reference to model the CPR artifact. They proposed two dual-channel methods, a Kalman state-space filter [43], and a filter based on the Gabor transform (time-frequency analysis) of the corrupted ECG and the reference signal [44]. These filters were optimized using mixtures of CPR artifacts recorded in pigs with 14 human VF samples. CPR was manually delivered at a rate of 80 cpm. Furthermore, Werther et al. [45] presented a comprehensive comparative assessment of these filters extending their rhythm database to 104 shockable and 281 nonshockable rhythms (other than asystole). Werther et al. compared the performance of four filters in a dual-channel configuration based on the blood pressure signal: their Kalman and Gabor filters, the MC-RAMP filter [41], and a recursive least squares (RLS) filter [46]. They tuned the filters for maximum SNR improvement and analyzed the performance of a SAA in terms of both sensitivity and specificity. All filters showed a comparable performance with good sensitivities, above 95%, but with specificities below 90%, caused by the higher spectral overlap of nonshockable rhythms with the CPR artifact. Later, Granegger et al. [47] applied independent component analysis (ICA) to 8 leads recorded in the surface of a dead pig after injecting human emergency ECGs close to the heart of the pig. Their database, which is fully described in [48], comprised 431 shockable and 487 nonshockable (20 asystole) records, with CPR delivered manually according to the 2005 guidelines. After applying ICA, they obtained a sensitivity of 99.7% and a specificity of 83.2% using the SAA of a commercial AED. These results marginally improved those obtained on the same data for the MC-RAMP filter using the force as reference [47]. Furthermore, a multilead configuration is not available in an AED environment.

Efforts have been made to adaptively filter the CPR artifact based only on the ECG because reference signals other than the thoracic impedance may not be available in AEDs. In these methods the fundamental frequency and harmonic content of the artifact are obtained from the spectral analysis of the corrupted ECG. These characteristics are then used to fit the adaptive filter, with solutions like an adaptive notch filter [49], a Kalman filter [27], or the coherent line removal algorithm [50]. Aramendi et al. [49] and Ruiz de Gauna et al. [27] introduced two methodological innovations by considering mixtures of shockable rhythms with CPR artifacts recorded from OHCA patients in asystole and by optimizing filter performance in terms of the sensitivity after filtering. In addition, Ruiz de Gauna et al. [45] used the mixture model to optimize their algorithm and reported their final results for human cardiac arrest data recorded during CPR.

However, adaptive filters based only on the ECG have poorer performance than adaptive filters using reference signals [27].

In summary, the mixture model is an excellent signal processing framework to test filter performance in terms of improvements in SNR and can serve well to optimize the parameters of a filter. However, SNR in real cardiac arrest data is not known, and how improvements in SNR are translated to the more clinically relevant sensitivity/specificity figures is not well understood [51] and may depend greatly on the SAA used. Finally, CPR may modify the dynamics of the underlying rhythm which violates the fundamental assumption of the independence of the ECG and the CPR artifact.

### 3.2. Studies Based on Cardiac Arrest Data Recorded during CPR

The limitations of the mixture model can be overcome using cardiac arrest data recorded while delivering CPR. During chest compressions the underlying rhythm is not directly observable, so these data are annotated by expert clinicians by assessing the rhythm in the intervals right after CPR and assuming the same rhythm for the preceding interval. Figure 1 shows three examples of these type of data: a VF, a PEA, and an asystole. Researchers then use short rhythm intervals (10–15 s) during CPR to optimize and test their rhythm analysis methods in terms of sensitivity and specificity. In this framework, rhythm analysis during CPR has been approached in two ways: adaptive filters followed by a SAA designed to diagnose artifact-free ECGs and new SAAs that directly analyze the corrupted ECG.

Most works covered in this section are based on human data, although a study by Berger et al. [46] investigated filtering schemes using an animal model of cardiac arrest. They induced asystole and VF in 13 pigs under normal sinus rhythm and delivered CPR to the pigs through a mechanical device (Zoll AutoPulse), which worked at a constant rate of 80 cpm [52]. They used an adaptive RLS filter based on the force signal provided by the compression device and analyzed the performance of three commercial AEDs. In these favorable conditions, porcine VF and low compression rates, they obtained a mean sensitivity and specificity of 97% and 95%, respectively, for 13 normal sinus rhythms, 8 asystole, and 109 VF records.

In 2004, Eilevstjønn et al. [14] published the first study that analyzed an adaptive filter to suppress the CPR artifact on recordings from OHCA victims. The study was based on data recorded in a clinical study [9] using a commercial defibrillator modified to acquire several additional reference signals, including those from a device to monitor CPR quality based on accelerometers. Eilevstjønn et al. adapted the MC-RAMP filter introduced by Husøy et al. [36] and used four reference signals to model the artifact: the thoracic impedance, the ECG common mode, the compression acceleration and the compression depth. Their database contained 184 shockable rhythms and 348 nonshockable rhythms randomly split into a training and a test set. After filtering, they obtained an excellent sensitivity of 96.7% but a low specificity of 79.9%.

Researchers then focused on reducing or eliminating the need for additional reference signals, in an effort to adapt these methods to a realistic AED scenario. (Some of these studies were based on the mixture model and are described in Section 3.1.) The Kalman filter based only on the ECG proposed by Ruiz de Gauna et al. [27] was tested on 131 shockable and 347 nonshockable rhythms extracted from the same original study used by Eilevstjønn et al. [14]. However, the overall results were poorer, 90.1% sensitivity and 80.4% specificity. Their results underlined the importance of using additional reference information to model the CPR artifact.

Using a dual-channel approach, Irusta et al. [15] proposed a CPR artifact model based on a time-varying Fourier series representation, which could be built using only the instantaneous frequency of the chest compressions. They obtained this frequency from the compression depth signal and adjusted the time-varying Fourier coefficients using a least mean squares (LMS) filter. The LMS filter was tested on 89 shockable and 292 nonshockable rhythms, with a sensitivity and specificity of 95.6% and 85.6%, respectively. Using this same database, Ruiz et al. [53] fitted the time-varying Fourier series model of the artifact by means of a Kalman filter. Furthermore, they conducted a spectral analysis of the rhythms and the CPR artifact and proved that the spectral overlap was larger for nonshockable rhythms, particularly for PEA. Aramendi et al. [28] showed that the instantaneous frequency used by the LMS filter could be derived from the thoracic impedance signal which is recorded by current AEDs through the defibrillation pads. This would eliminate the need of a chest device for acquiring additional reference signals. Finally, Ruiz de Gauna et al. [54] used an LMS finite impulse response filter to estimate the artifact using the force signal, in an effort to replicate the good results reported by Berger et al. [46] for a porcine model. The method was tested on 88 shockable and 292 nonshockable records; the sensitivity was 95.5% but the specificity after filtering was only 86.6%.

Tan et al. [29] introduced their artifact reduction and tolerant (ART) adaptive filter, which is currently integrated in a commercial AED (See-Thru CPR, ZOLL Medical), as a clinical support tool. Their adaptive filter is based on the CPR sternal velocity signal obtained by this particular AED from an accelerometer incorporated to the defibrillation pads which is placed beneath the rescuers hand. When tested on 114 shockable and 4155 nonshockable rhythms the method showed a sensitivity of 92.1% and a specificity of 90.5%.

In addition to adaptive filters, methods based on the direct analysis of the corrupted ECG have also been explored. In 2008, Li et al. [30] presented the first rhythm analysis method to directly diagnose the ECG corrupted by CPR artifacts, which was based on an ECG feature that is marginally affected by the artifact. This feature was obtained from the wavelet transform and the correlation function. The algorithm was validated with 1256 shockable and 964 nonshockable rhythms recorded from 229 OHCA patients during CPR, yielding a sensitivity of 93.3% and a specificity of 88.6%. Their method was proved to be more reliable for VF detection in the presence of CPR artifacts than several classical VF detection methods [55]. More recently, Krasteva et al. [31] presented a second method, this time based on features derived from the corrupted ECG and a reconstructed version of the ECG. After optimization, Krasteva et al. tested their algorithm on 172 shockable and 721 nonshockable rhythms obtained from 100 OHCA patients, for a sensitivity of 90.1% and a specificity of 86.1%.

Table 1 summarizes the results reported by six representative methods for rhythm analysis during CPR tested on human cardiac arrest data. The results cannot be directly compared for two reasons. First, the studies are based on different data, with very different prevalence of the rhythm types and different selection criteria for the rhythms. For example, these studies have large differences in the proportion of asystole among nonshockable rhythms, which may have important implications in the results given that asystole is the nonshockable rhythm with the largest prevalence [56] and the main cause of the low specificity [27]. Second, the studies based on adaptive filtering use different SAAs that may diagnose the filtered ECG differently. In fact, adaptive filters have been shown to have very similar sensitivities and specificities when tested using the same data and the same SAA [45, 57].

In any case, all these studies have some common limitations. Although the sensitivity is good, all studies present specificities well below the 95% recommended by the AHA. This would result in a large number of erroneous shock diagnoses during CPR, which would cause unnecessary CPR interruptions for nonshockable rhythms. In addition, these methods are evaluated using short rhythm intervals (10–20 s), which are sufficient for a shock/no-shock diagnosis and an evaluation of the method in terms of sensitivity and specificity. However, rhythm analysis during CPR is conceived to continuously diagnose the rhythm with the objective of improving CPR delivery compared to the standard CPR protocol, which requires interrupting CPR every two minutes for rhythm analysis. In this scenario the methods must be evaluated using long duration records, and a new methodology that goes beyond sensitivity/specificity for a single analysis is needed to quantify the effect of using these methods on the delivery of CPR. Over the last year, some studies have addressed and partially overcome these limitations. The following two sections describe these late advances in detail.

## 4. Rhythm Analysis during CPR: Impact on CPR Delivery

Current CPR guidelines recommend 2 minutes of uninterrupted CPR followed by a pause for rhythm reassessment [12, 13]. Rhythm analysis methods during CPR are conceived to improve CPR delivery compared to these recommendations. In this context, a rhythm analysis method would continuously analyze/monitor the rhythm during CPR with two objectives. First, advancing the shock to patients with shockable rhythms, which could be beneficial given the high oxygen demands of recurrent VF [58]. Second, prolong uninterrupted CPR beyond two minutes for patients with nonshockable rhythms, therefore increasing the chest compression fraction which increases the likelihood of ROSC [11].

In 2005, Eilevstjønn et al. [59] proposed a set of modifications in AED operation to potentially reduce no-flow times (NFT), which is equivalent to increasing the chest compression fraction. These modifications included continuous rhythm analysis during CPR and, in the event of a shockable rhythm, a short hands-off period for rhythm verification in which the capacitor would also be charged. In addition, they proposed 1 min of uninterrupted CPR immediately after a shock and rhythm analysis during CPR starting after that minute. They analyzed 105 complete resuscitation episodes and concluded that the median NFT could be theoretically reduced from 51% to 34% and from 49% to 39% for patients in shockable and nonshockable rhythms, respectively. Eilevstjønn et al. did not consider the impact of misdiagnosing the rhythm during chest compressions in their estimations of the potential reduction in NFT. However, errors in diagnosis would be frequent given the low specificity of current methods. Consequently, the real impact on CPR delivery of continuous rhythm analysis was not assessed.

**Figure 6:**
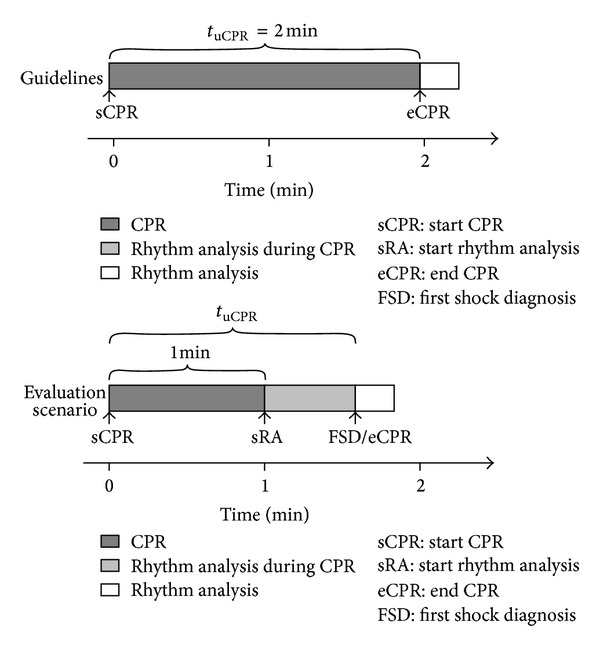
Evaluation scenario proposed by Ruiz et al. [24] for continuous rhythm analysis during CPR, which consists of 1 minute of uninterrupted CPR followed by rhythm analysis during CPR. CPR stops when the rhythm analysis method gives the first shock diagnosis. The *t*_uCPR_ obtained in this manner is then compared to the guideline's recommendation of 2 minutes of *t*_uCPR_ after a shock or a pause for rhythm reassessment. The figure has been adapted from Ruiz et al. [24].

Ruiz et al. [24] recently introduced a methodology to evaluate the real impact of rhythm analysis methods on CPR delivery. The methodology is based on the evaluation scenario described in Figure 6. This scenario starts with 1 minute of uninterrupted CPR, as introduced by Eilevstjønn et al. [59], to guarantee a minimum period of blood flow. Then rhythm analysis during CPR starts and CPR continues until a shock is advised. In this scenario, the time to the first shock diagnosis determines the duration of the uninterrupted CPR time (*t*_uCPR_). For an adaptive filter followed by a SAA, Ruiz et al. computed *t*_uCPR_ on 242 shockable and 634 nonshockable long duration OHCA segments. Then they estimated the probability of interrupting CPR as a function of time using Kaplan-Meier survival curves for both shockable and nonshockable rhythms.

The rhythm analysis method had a sensitivity of 94% and specificity of 81%, that is, an accuracy comparable to those reported in the literature. However the estimated impact on CPR delivery was much larger than anticipated. Although 100% of patients in shockable rhythms would receive a shock earlier, CPR would be interrupted before 2 minutes in 42% of patients in nonshockable rhythms. This would reduce the chest compression fraction in a large number of cases resulting in a compromised probability of survival.

Methodologically, the study by Ruiz et al. starts a new stage in rhythm analysis during CPR centered on evaluating the effects on CPR delivery of using these methods. Their results confirm and amplify a well known problem; the specificity of current methods is still too low. However, the impact of the low specificity on CPR delivery is much larger than anticipated. New strategies to reduce interruptions in CPR delivery are needed.

## 5. New Strategies to Rhythm Analysis during CPR

To date, the methods for rhythm analysis during CPR have focused mainly on two key ideas: (1) analyzing the rhythm during chest compressions and (2) prioritizing the detection of shockable records above the detection of nonshockable records. Unfortunately the accuracy of the methods has not improved much over these last years. Consequently, some recent efforts have started to explore new strategies for rhythm analysis during CPR.

### 5.1. Rhythm Analysis during Chest Compression Pauses

**Figure 7:**
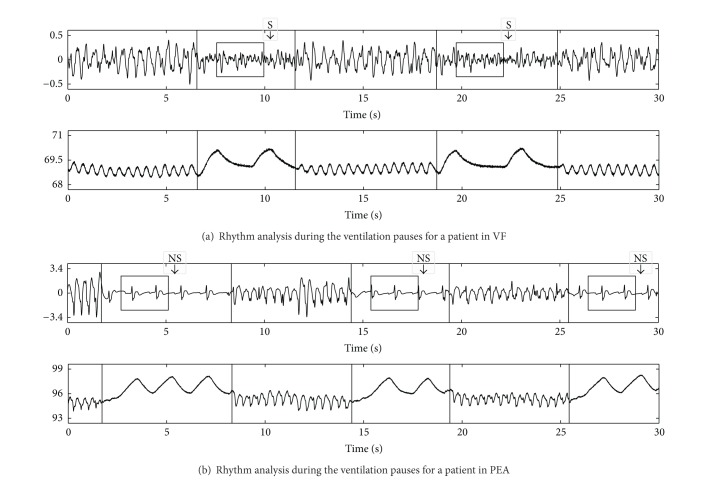
Examples of rhythm analysis during the ventilation pauses; in both examples the top panels show the ECG in mV and the lower panel shows the thoracic impedance in *Ω*. In the impedance channel chest compression artifacts (fast fluctuations) and ventilation artifacts (slow fluctuations) are visible. During the pauses for ventilation there are no chest compression artifacts in the ECG and the high temporal-resolution SAA gives an accurate diagnosis using 3 s windows. The examples have been adapted from Ruiz et al. [25].

Before tracheal intubation current resuscitation guidelines recommend a 30 : 2 compression to ventilation (CV) ratio for CPR. Each cycle of 30 chest compressions, which at the standard rates takes approximately 18 s, is followed by a pause for two rescue breaths. Although the guidelines limit the time for two rescue breaths to 5 s, in real practice the median pause duration is 7 s [60]. During ventilations there are no visible artifacts that may affect rhythm analysis, as shown in Figure 7. Based on this premise, Ruiz et al. [25] proved that it was possible to analyze the rhythm during chest compression pauses, ventilation or nonventilation pauses, using a high temporal-resolution SAA, that is, an algorithm capable of giving an accurate diagnosis in 3 s [26]. Figure 7 illustrates this method for a shockable and a nonshockable rhythm. They analyzed 110 shockable and 466 nonshockable long duration OHCA segments and manually identified a total of 4476 pauses in chest compressions, of which 2183 were ventilation pauses with two rescue breaths. The pauses had a median duration of 6.1 s, 5.5 s for those with two rescue breaths, and 91% of all the pauses and 95% of the ventilation pauses with two breaths were longer than 3 s, which made them suitable for a rhythm analysis by the SAA. The sensitivity and specificity were 95.8% and 96.8%, respectively, well above the AHA recommendations.

A key component to incorporate this solution into a defibrillator is the automatic identification of the intervals without chest compressions. Depending on the available equipment, different reference channels could be used for this purpose. In a scenario with an external CPR assist device the identification could be performed using the compression depth or the force channels. However, most defibrillators do not incorporate this technology, so a more general solution based on the impedance signal should be explored. Pauses in chest compressions [61], ventilations [62], and the end of chest compressions [63] have already been detected on the impedance, although a complete valid system has not yet been demonstrated.

Devices incorporating this solution would have an accurate rhythm analysis approximately every 18 s for CPR delivered at a 30 : 2 CV ratio for a standard compression rate of 100 cpm. The AED could then guide therapy using this feedback to monitor nonshockable rhythms or for early recognition of recurrent VF, converting AEDs into intelligent devices.

### 5.2. Rhythm Analysis during Chest Compressions

In the last years there has been an increasing debate about the need for active ventilations during CPR. Several studies have shown an increased survival rate when compression only CPR (COCPR) was administered compared with the standard 30 : 2 CV ratio CPR [64, 65]. In the future resuscitation guidelines may recommend COCPR. In fact, current guidelines state that COCPR may be used by untrained bystanders or bystanders unwilling to give rescue breaths [12, 13]. In this scenario, new and reliable methods to analyze the rhythm during chest compressions should be developed.

As shown in Section 4, in a continuous rhythm analysis scenario CPR would only be stopped when a shock is advised. If the patient presents a shockable rhythm, an erroneous no-shock diagnosis could be corrected in the upcoming rhythm analyses if the sensitivity of the method is not too low. On the other hand, for patients in nonshockable rhythms a single erroneous shock diagnosis entails an unnecessary CPR interruption. Consequently, efforts should focus on increasing the specificity. Based on our 10-year experience on this field, we believe that the following three strategies should be explored and combined.

1. From a SAA design perspective the accuracy of the method could be increased by merging the two most successful strategies for rhythm analysis during CPR: adaptive filters to suppress the CPR artifact combined with rhythm analysis algorithms designed to work during CPR. Although adaptive filters substantially reduce the CPR artifact, with SNR improvements of up to 35 dB [29], a filtering residual always remains. These residuals frequently resemble a disorganized rhythm [14, 15, 53] and may produce a shock diagnosis in SAAs designed for artifact free ECGs. This is particularly severe when the underlying nonshockable rhythm has low electrical activity like during asytole or low rate PEA. SAAs designed to analyze the ECG in the presence of filtering residuals should be designed with emphasis on increasing the specificity.
2. Sometimes the chest compression artifact is so large that even state of the art adaptive filters cannot effectively eliminate it. In these cases the rhythm analysis following filtering is grossly equivalent to a coin toss. However, if the rhythm is continuously analyzed these unreliable analyses can be safely ignored until the amplitude of the artifact decreases. SAAs could add a block before rhythm analysis to identify large chest compression artifacts and wait until a safe rhythm analysis is possible.
3. The confidence in a shock decision could be further increased by efficiently combining several rhythm analysis decisions. For instance, instead of using a shock/no-shock decision per analysis window, the algorithm could return an estimate of the probability of having a shockable rhythm. In a continuous rhythm analysis scenario several of these probabilities could be conservatively combined before a shock is actually decided.

Rhythm analysis during CPR could be further enhanced if these strategies were combined with techniques to determine the optimal time for shock delivery. In the past 20 years, considerable efforts have been made on VF waveform analysis to define predictors of defibrillation success and outcome such as median slope [66], scaling exponent [67], and amplitude Spectrum Analysis (AMSA) [68, 69]. Incorporating rhythm analysis during CPR and assessment of the optimal time to defibrillate would lead to a new generation of intelligent AEDs, capable of guiding therapy individually.

Finally, rhythm analysis methods during chest compressions should be evaluated in terms of their impact on CPR delivery, as described in Section 4. Ruiz et al. [24] proposed that for nonshockable rhythms these methods should guarantee a probability greater than 95% of delivering at least 2 minutes of uninterrupted CPR (meet guidelines) and a probability greater than 90% of delivering at least 3 minutes of uninterrupted CPR (improve chest compression fraction compared to guidelines). In addition, they should guarantee that the shock is advanced in at least 90% of shockable rhythms. Although these recommendations seem reasonable, they should be appraised by the resuscitation research community.

## 6. Conclusions

Currently, there is insufficient evidence to support or refute the use of algorithms for rhythm analysis during CPR. The evaluation of these algorithms in terms of sensitivity and specificity on short ECG segments does not accurately predict their impact on CPR delivery. As stated by the CoSTR, studies must demonstrate that rhythm analysis during CPR optimizes the time of appropriate chest compressions. To this aim, the probability of interrupting CPR as a function of time has been proposed as a new evaluation figure. In this new framework, the classical sensitivity/specificity goals would change to new goals for uninterrupted CPR time.

Recently, new solutions have been proposed for rhythm analysis during CPR. Hands-off intervals for rhythm analysis could be completely eliminated by assessing the rhythm during ventilation pauses using a high temporal-resolution SAA. On the other hand, accurate SAAs with high specificity should be designed to work during chest compressions for COCPR scenarios. Retrospective studies with large databases of complete OHCA episodes should be conducted to simulate continuous rhythm analysis and measure the impact on CPR delivery. Later, prospective studies using defibrillators incorporating these algorithms could definitely prove if survival improves.

## Figures and Tables

**Table 1:** Comparison of six different approaches to rhythm analysis during CPR tested on OHCA registers. The confidence intervals for sensitivity (Se) and specificity (Sp) were computed using Wald's interval for binomial proportions. For the number of nonshockable rhythms the proportion is indicated in parenthesis, and NA stands for not available.

Authors	Method	Se (%)	Sp (%)	Testing datasets	
				S	NS
Eilevstjønn et al. [14]	MC-RAMP	96.7 (87.6–98.0)	79.9 (73.3–85.2)	92	174 (30%)
Ruiz de Gauna et al. [27]	Kalman filter	90.1 (83.6–94.2)	80.4 (75.9–84.3)	131	347 (43%)
Aramendi et al. [28]	LMS filter	95.4 (88.4–98.6)	86.3 (81.8–89.9)	87	285 (31%)
Tan et al. [29]	ART filter	92.1 (86.8–95.5)	90.5 (89.7–91.2)	114	4155 (NA)
Li et al. [30]	Direct analysis	93.3 (92.0–94.4)	88.6 (86.8–90.2)	1256	964 (4%)
Krasteva et al. [31]	Direct analysis	90.1 (85.6–94.6)	86.1 (83.6–88.7)	172	721 (46%)
